# Integrase Inhibitor Prodrugs: Approaches to Enhancing the Anti-HIV Activity of β-Diketo Acids

**DOI:** 10.3390/molecules200712623

**Published:** 2015-07-13

**Authors:** Vasu Nair, Maurice Okello

**Affiliations:** Center for Drug Discovery and College of Pharmacy, University of Georgia, Athens, GA 30602, USA; E-Mail: mokello@uga.edu

**Keywords:** prodrugs, HIV-1 integrase inhibitors, antiviral activity

## Abstract

HIV integrase, encoded at the 3′-end of the HIV pol gene, is essential for HIV replication. This enzyme catalyzes the incorporation of HIV DNA into human DNA, which represents the point of “no-return” in HIV infection. Integrase is a significant target in anti-HIV drug discovery. This review article focuses largely on the design of integrase inhibitors that are β-diketo acids constructed on pyridinone scaffolds. Methodologies for synthesis of these compounds are discussed. Integrase inhibition data for the strand transfer (ST) step are compared with *in vitro* anti-HIV data. The review also examines the issue of the lack of correlation between the ST enzymology data and anti-HIV assay results. Because this disconnect appeared to be a problem associated with permeability, prodrugs of these inhibitors were designed and synthesized. Prodrugs dramatically improved the anti-HIV activity data. For example, for compound, **96**, the anti-HIV activity (EC_50_) improved from 500 nM for this diketo acid to 9 nM for its prodrug **116**. In addition, there was excellent correlation between the IC_50_ and IC_90_ ST enzymology data for **96** (6 nM and 97 nM, respectively) and the EC_50_ and EC_90_ anti-HIV data for its prodrug **116** (9 nM and 94 nM, respectively). Finally, it was confirmed that the prodrug **116** was rapidly hydrolyzed in cells to the active compound **96**.

## 1. Introduction

HIV-1 integrase, which is encoded at the 3′-end of the *pol* gene of the human immunodeficiency virus (HIV), is a retroviral enzyme, which is required for the replication of HIV. Because of the critical nature of the biochemical step that it facilitates, *i.e.*, the incorporation of viral DNA into human chromosomal DNA, which is viewed as the “point of no-return” in HIV infection, it is a significant target for the discovery of anti-HIV drugs [1,2,3,4,5,6,7,8,9,10,11]. Research efforts in the area of anti-HIV integrase inhibitors for the treatment of acquired immunodeficiency syndrome (AIDS) has resulted in three compounds—raltegravir, elvitegravir, and dolutegravir—that have been approved by the FDA for the clinical treatment for HIV-AIDS [7,8,9,10]. However, as toxicological issues, drug interaction problems and emerging resistance are common and recurring issues with all targeted classes of anti-HIV drugs, the discovery of anti-HIV active integrase inhibitors continues to be of significant scientific importance.

The 32 kDa retroviral integrase of HIV-1 [1,12,13] catalyzes the insertion of HIV DNA into host DNA by utilizing a 3′-processing (3′P) step and a strand transfer (ST) step. The 3′P step, which is the initial step after the binding of integrase with viral cDNA previously produced by reverse transcription, takes place in the cytoplasm [1,3,12,13,14,15]. The processed intasome (*i.e.*, truncated viral DNA-integrase complex) is then transported into the nucleus where the ST step takes place.

**Figure 1:**
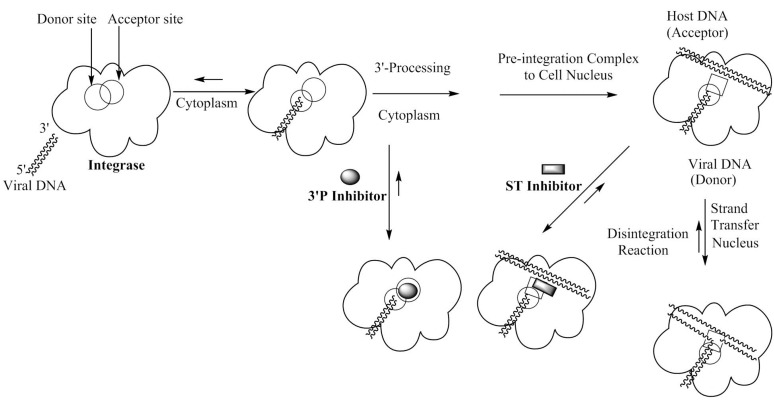
Mechanistic representation of the possible biochemical point(s) of inhibition of integrase (*i.e.*, 3′-processing and/or strand transfer) by an inhibitor [3]. Most integrase inhibitors are targeted at the strand transfer step in the nucleus.

A suggested mechanism for the inhibition of HIV integrase is shown in Figure 1 [3,5,16]. In the initial stage, which occurs in the cytoplasm, there is recognition and binding of HIV integrase to viral DNA. This is facilitated through recognition by integrase of specific sequences in the long terminal repeats (LTRs) of viral DNA. The first step in the integration process is 3′P in which there is site specific endonuclease activity and two nucleotide units are removed from each end of double-helical viral DNA. After the 3′P step, the multimeric pre-integration complex of tailored viral DNA and integrase is transported through the nuclear envelope and into the nucleus where integrase catalyzes the insertion of the processed viral DNA ends into host chromosomal DNA. This ST step involves staggered nicking of chromosomal DNA and subsequent joining of each 3′-end of the recessed viral DNA to the 5′-ends of the host DNA. The joining produces a gapped intermediate and integration is then completed through repair/ligation. Divalent metal ion cofactors are required for both 3′P and ST steps. Inhibitors of the two key steps of integration act via chelation of the metal cofactors necessary for the enzyme activity. As shown in Figure 1, inhibition can occur in the cytoplasm and/or in the nucleus.

In this review, we report on the discovery of HIV integrase inhibitors constructed with a heterocyclic scaffold adorned with hydrophobic groups and a 2-hydroxy-4-oxo-but-2-enoic acid multifunctional component. We also report on enhancement of the anti-HIV activity of these compounds through prodrug synthesis. Supporting data is presented that provide unmistakable evidence that the antiviral activities of these compounds can be improved through conversion to prodrugs. There has been considerable interest over the years in the development of prodrugs in the treatment of HIV/AIDS with a view to increasing the permeability of the drugs and minimizing undesirable side effects. A representative example is the acyclic phosphonate, tenofovir, for which its prodrug, tenofovir disoproxil fumarate, is the administered drug for use in HIV combination therapeutic applications [17,18,19,20]. Also of relevance is a recent report on the development of raltegravir-based prodrugs to enhance colonic absorption and to lower frequency of administration [21,22].

## 2. Discovery of Small Molecule Inhibitors of HIV-1 Integrase

### 2.1. Beta-Diketo Acids as HIV Integrase Inhibitors

While many structurally diverse compounds have been reported to be inhibitors of HIV integrase [5,6,23,24,25,26,27,28,29,30,31,32,33,34,35,36], the compounds of one group, the β-diketo acids and their congeners and related compounds, are among some of the best known, biologically-validated inhibitors of this key viral enzyme [30,31,37]. Some representative examples of these compounds are identified in the tables that follow (see Table 1, Table 2, Table 3, Table 4 and Table 5). We will focus on some of the more active compounds of this group with respect to integrase inhibition enzymology data, anti-HIV activity in cell culture, pre-clinical data and clinical trial results. It should be noted that integrase inhibition enzymology data may vary to some extent depending on whether MnCl_2_ was substituted for MgCl_2_ in these assays, but the differences in data are usually not large [16].

#### Integrase Inhibition Protocol

Integrase inhibition studies on the presented compounds were conducted with recombinant wild-type HIV-1 integrase and a 21-mer oligonucleotide substrate following a previously described procedure, an example of which is summarized below [25,38,39].

All study compounds were dissolved in DMSO and the stock solutions were stored at −20 °C. The γ [^32^P]-ATP was purchased from either Amersham Biosciences or ICN. The expression systems for the wild-type IN and soluble mutant INF185KC280S were generous gifts of Dr. Robert Craigie, Laboratory of Molecular Biology, NIDDK, NIH, Bethesda, MD. The oligonucleotides 21top, 5′-GTGTGGAAAATCTCTAGCAGT-3′ and 21bot, 5′-ACTGCTAGAGATTTTCCACAC-3′ were purchased from Norris Cancer Center Micro-sequencing Core Facility (University of Southern California, Los Angeles, CA, USA) and purified by UV shadowing on polyacrylamide gel. To analyze the extent of 3′-processing and strand transfer using 5′-end labeled substrates, 21top was 5′-end labeled using T4 polynucleotide kinase (Epicentre, Madison, WI, USA) and γ [^32^P]-ATP (Amersham Biosciences or ICN). The kinase was heat-inactivated and 21bot was added in 1.5-molar excess. The mixture was heated at 95 °C, allowed to cool slowly to room temperature, and run through a spin 25 mini-column (USA Scientific, Ocala, FL, USA) to separate annealed double-stranded oligonucleotide from unincorporated material. To determine the extent of 3′-processing and strand transfer, wild-type IN was preincubated at a final concentration of 200 nM with the inhibitor in reaction buffer (50 mM NaCl, 1 mM HEPES, pH 7.5, 50 µM EDTA, 50 µM dithiothreitol, 10% glycerol (*w*/*v*), 7.5 mM MnCl_2_, 0.1 mg/mL bovine serum albumin, 10 mM 2-mercaptoethanol, 10% dimethyl sulfoxide, and 25 mM MOPS, pH 7.2) at 30 °C for 30 min. Then, 20 nM of the 5′-end ^32^P-labeled linear oligonucleotide substrate was added, and incubation was continued for an additional one hour. Reactions were quenched by the addition of an equal volume (16 µL) of loading dye (98% deionized formamide, 10 mM EDTA, 0.025% xylene cyanol and 0.025% bromophenol blue). An aliquot (5 µL) was electrophoresed on a denaturing 20% polyacrylamide gel (0.09 M trisborate pH 8.3, 2 mM EDTA, 20% acrylamide, 8 M urea). Gels were dried, exposed in a PhosphorImager cassette, and analyzed using a Typhoon 8610 Variable Mode Imager (Amersham Biosciences, Piscataway, NJ, USA) and quantitated using ImageQuant 5.2. Percent inhibition (% I) was calculated using the following equation: % I = 100 × [1 − (D − C)/(N − C)], where C, N, and D are the fractions of 21-mer substrate converted to 19-mer (3′P product) or ST products for DNA alone, DNA plus IN, and IN plus drug, respectively. The IC_50_ values were determined by plotting the logarithm of drug concentration *vs.* percentage inhibition to obtain a concentration that produced 50% inhibition.

### 2.2. Anti-HIV Activity Protocols

All antiviral determinations were performed in triplicate with serial ½log10 dilution of the test materials (six to nine concentrations total). The overall performance of both assays was validated by the MOI-sensitive positive control compound, AZT, which exhibited the expected level of antiviral activity [4].

#### 2.2.1. Anti-HIV Evaluation in Fresh Human PBMCs

Study compounds and the control compound, AZT, were tested in a PBMC cell-based, microtiter anti-HIV assay against the clinical isolate, HIV-1TEKI (NSI phenotype) and HIV-1_NL4-3_ (SI phenotype). Low-passage, lymphotropic clinical isolate, HIV-1TEKI, was obtained from a pediatric patient. HIV-1_NL4-3_ was generated via transfection of pHIV-1_NL4-3_ plasmid DNA into HeLa cells and collecting infectious virus from the tissue culture supernate. Fresh human PBMCs were isolated from screened donors, seronegative for HIV and HBV and processed in the established way for these studies. For the standard PBMC assay, PHA-P stimulated cells from at least two normal donors were pooled (to minimize variability), diluted and plated in 96-well microplates. Each plate contained virus control wells (cells plus virus) and experimental wells (drug plus cells plus virus). Parallel drug cytotoxicity studies (without virus) used an MTS (Promega) assay system. Following infection, the PBMC cultures were maintained for seven days at 37 °C, 5% CO_2_. After this period, cell-free supernatant samples were collected for analysis of reverse transcriptase activity and cells were stained with MTS to determine compound cytotoxicity. Wells were also examined microscopically for any abnormalities.

#### 2.2.2. Anti-HIV Assays in MAGI Cells

In MAGI-X4 cell assay, the MAGI cells were infected with HIV-1_NL4-3_ in the presence of test compound. If the virus is able to infect and replicate in the cells it will proceed through reverse transcription and integration and begin transcription from the integrated provirus. One of the first virus proteins produced is HIV-1 Tat, which transactivates the HIV-1 LTR promoter driving expression of β-galactosidase from an LTR-β-galactosidase reporter gene construct engineered into the cells. As a result, infected cells begin to overproduce the β-galactosidase enzyme. Forty-eight hours (single-cycle assay) or 6 days (multi-cycle assay) post infection, the cells are lysed and β-galactosidase enzyme activity is measured using a chemiluminescence detection method (Perkin Elmer Applied Biosystems, Waltham, MA, USA). Compound toxicity is monitored on replicate plates using MTS dye reduction (CellTiter 96^®^ Reagent, Promega, Madison, WI, USA).

#### 2.2.3. Anti-HIV Assays in GHOST X4/R5 Cells

Twenty-four hours prior to inititiation of the assays, the cells were trypsinized, counted and 2 × 10^4^ GHOST X4/R5 cells/well placed in media without selection antibiotics. At 24 h, media was removed and compound in media was placed on the cells. A known titer of HIV-1_NL4-3_ or HIV-1_TEKI_ was then added to the wells and and the cultures were incubated for seven days at 37 °C. At termination of the GHOST X4/R5 assay, cell-free supernatant samples were collected for analysis of viral reverse transcriptase activity. Compound toxicity was monitored on uninfected sister plates by MTS dye reduction. All determinations were performed in triplicate with serial ½log_10_ dilution of test materials.

### 2.3. Anti-HIV Activity of Selected Aryl and Heteroaryl Diketo Acid Integrase Inhibitors

An early example of a diketo acid with HIV-1 integrase inhibitory activity was the aromatic diketo acid, **1** (**L-708,906**, Table 1) [26,34,40,41]. Enzymology data revealed it to be a selective inhibitor of the ST step of integrase with an IC_50_ of 0.1 μM [42]. The compound also exhibited *in vitro* anti-HIV activity in HIV-1 infected H9 cells with an EC_50_ of 2.0 μM. The monobenzyl analogue, **2**, also showed anti-HIV activity [36]. All of the other compounds with aromatic scaffolds (**3**–**7**, Table 1) were also selective for inhibition of the ST step and exhibited *in vitro* anti-HIV activity at the µM level. Cell cytotoxicity did not appear to be an issue. Compounds in which the diketo acid moiety is attached to a heterocyclic system (**8**–**11**) were also found to have ST inhibitory activity and a few of these compounds were found to be anti-HIV active, but again at the µM level. Perhaps, the best known compound of the series of compounds listed in Table 1 is **11** (S-1360), which inhibited HIV-1 integrase with the enzymology-based IC_50_ of 0.02 μM. [32,33]. Anti-HIV assays in PBMC cells showed that compound **11** had good activity (EC_50_ 0.14 μM; CC_50_ 110 μM), displaying a therapeutic index (TI) of 786. In *in vitro* studies in cytosol, the major metabolite was the NADPH-dependent reduction of the enolic group through aldo-keto reductases [43]. This pathway may provide a mechanism for the clearance of compound **11**. However, pharmacokinetic data from phase II clinical trials revealed very low plasma concentrations of **11** in a majority of subjects. This is likely due to the rapid reduction of **11** followed by UGT-mediated glucuronidation and clearance of the glucuronide. For this reason, the development of **11** (S-1360) by Shionogi and Co. and GlaxoSmithKline was discontinued.

An overall analysis of the integrase inhibition and *in vitro* anti-HIV activity data in Table 1 revealed an irregular disconnect in much of the data. As an example, **8** (**L-731,988)** showed very good activity in inhibition of the ST step (IC_50_ 170 nM), but the anti-HIV data (EC_50_ 1.0 µM) was almost six times higher. This disconnect with **8** (**L-731,988)** was also observed with **11** (S-1360),which showed a seven-fold higher EC_50_ data compared to the ST IC_50_.

**Table 1:** Selected aryl or heteroaryl diketo acid integrase inhibitors.

Compound	(ST) IC_50_, μM *	Anti-HIV Data EC_50_ or EC_95_ (μM) * and Cell Line	Cytotoxicity CC_50_ or CC_95_ (μM) * and Therapeutic Index (TI) *	References
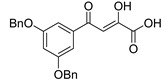 **1** **L-708,906**	0.10 3.5	EC_50_, 2.0 H-9 Cells EC_50_, 5.5 MT-4 Cells	CC_50_, 88.3 (TI = 16)	[26,34]
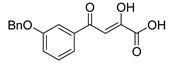 **2**	0.35 Mn^2+^	EC_50_, 0.6 293T Cells	-	[36]
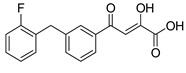 **3**	<0.10	EC_95_, 0.52 MT-4 Cells	-	[27]
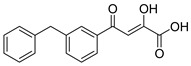 **4**	<0.10	EC_95_, 1.11 MT-4 Cells	CC_95_, >50 (TI > 45)	[27]
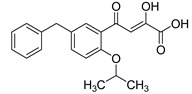 **5**	<0.10	EC_95_, 0.10 MT-4 Cells	CC_95_, >50 (TI > 500)	[27]
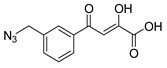 **6**	1.53 Mn^2+^	EC_50_, 2.1 293T Cells	CC_50_, >50 (TI > 24)	[27]
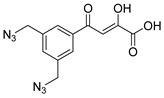 **7**	2.4 Mn^2+^	EC_50_, 5 293T Cells	CC_50_, >50 (TI > 10)	[44]
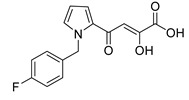 **8** **L-731,988**	0.17	EC_50_, 1.0 H-9 EC_95_, 9.6 MT-4 Cells	-	[26,27]
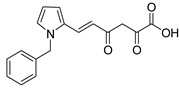 **9**	7.0	EC_50_, 1.5 MT-4/KB Cells	61 TI = 41	[45]
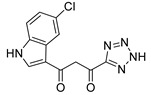 **10** **5ClTEP**	0.65 Mn^2+^	-	-	[36]
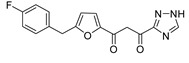 **11** **(S-1360)**	0.02	EC_50_, 0.14 PBMC	CC_50_, 110 (TI = 786)	[32,33]

* IC_50_ is the concentration for 50% inhibition of the specified step of integrase activity. EC_50_ is the concentration for 50% inhibition of virus replication. EC_95_ is the concentration for 95% inhibition of virus replication. CC_50_ is the concentration to reduce cell viability by 50%. CC_95_ is the concentration to reduce cell viability by 95%. TI (therapeutic index) is the ratio of CC_50_ to EC_50_.

### 2.4. HIV Integrase Inhibitors with Bis-Diketo Acid Structures

**Figure 2:**
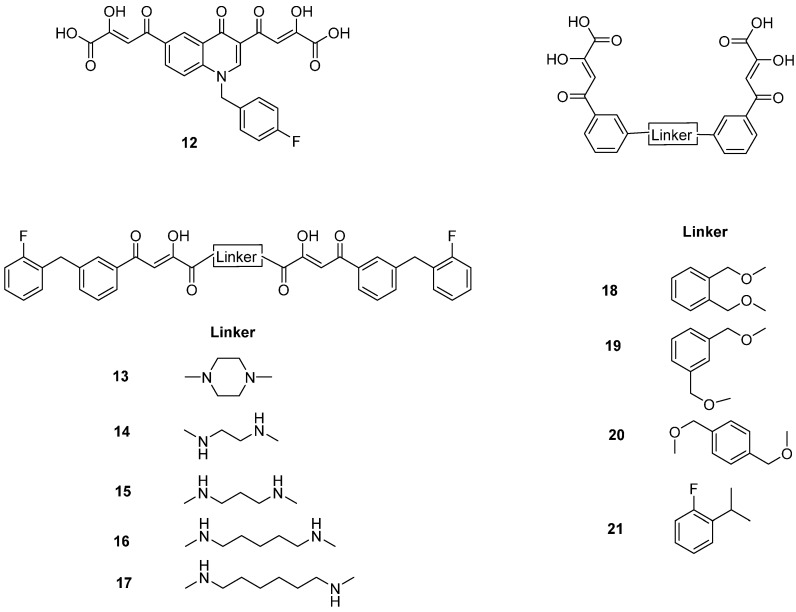
Structures of representative bis-diketo acids.

In Figure 2, the structures of HIV integrase inhibitors with two diketo acid components that are tethered with a variety of linking groups are shown. The intent of this work was to examine the ability of each diketo acid group in these compounds to bind to divalent metal ions that are critical for one or both steps of the mechanism of action of HIV integrase. Implicit in this drug design was the expectation that these compounds would have greater anti-HIV activity than their simpler monofunctional systems. The results of integrase inhibitory activity and anti-HIV activity data are summarized in Table 2 [46,47,48]. While one of the compounds (**12**) showed notable ST inhibitory activity, the remaining compounds exhibited marginal integrase inhibition. The *in vitro* anti-HIV activity data and therapeutic indices of all of the compounds listed in Table 2 were not compelling.

**Table 2:** Inhibitors of wild type HIV-1 integrase that are bis-diketo acids [46,47].

Compound	3′P (IC_50_, μM) * Mn^2+^ Assay	ST (IC_50_, μM) Mn^2+^ Assay	Anti-HIV-1 Data EC_50_ (μM), * CEM-SS Cells (Except 12, H-9 Cells) uH	CC_50_ (μM) (TI = CC_50_/EC_50_) *
**12**	0.2	0.01	4.3	>200 (TI > 47)
**13**	2.5	0.3	0.8	11 (TI = 14)
**14**	5.8	0.2	>5	5 (TI < 1)
**15**	5.3	0.2	>9	9 (TI < 1)
**16**	4.8	0.7	>12	12 (TI < 1)
**17**	32	2.7	16	124 (TI = 8)
**18**	1.8	0.3	39	>200 (TI > 5)
**19**	7.3	0.4	>200	>200 (TI = 1)
**20**	1.7	1.9	>200	>200 (TI = 1)
**21**	1.8	0.2	17	81 (TI = 5)

* IC_50_ is the concentration for 50% inhibition of the specified step of integrase activity. EC_50_ is the concentration for 50% inhibition of virus replication. CC_50_ is the concentration to reduce cell viability by 50%. TI (therapeutic index) is the ratio of CC_50_ to EC_50_.

### 2.5. Discovery of Diketo Carboxylic Acids with Nucleobase Scaffolds

**Figure 3:**
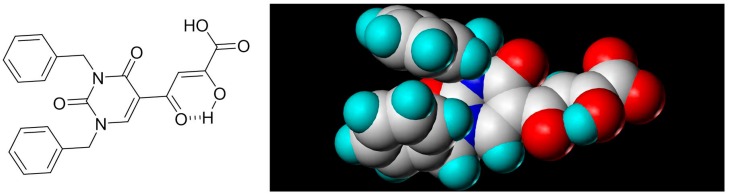
The novel integrase inhibitor, **22**, shown in its intramolecular hydrogen-bonded form (**left**), and the preferred conformation of its physiologically-relevant anion (**right**) depicting the relationship of the two benzyl groups and of the enolic hydrogen with the keto carbonyl [4].

In our laboratory, we discovered new β-diketo acids with nucleobase scaffolds that were potent inhibitors of both 3′-processing and ST steps of HIV integrase [4]. Our structure-activity data suggest that the nucleobase scaffold, the nature of substituents on the scaffold, and a specific spatial relationship of the substituents on the scaffold are critical for potent integrase inhibitory activity. The discovery made these compounds unique among diketo acids, not only in terms of integrase activity, but also because of their remarkably potent anti-HIV activity. The scaffolds involved examples of both pyrimidine and purine systems. The discovery can be appropriately illustrated with one notable example in which the structural features are as follows: a pyrimidine scaffold; three specific substituents in a defined spatial relationship with hydrophobic benzyl groups at N-1 and N-3 and a diketo acid group at C-5. Also, the enolic form of the diketone functionality is the major tautomer. The parent compound of the series was 4-(1,3-dibenzyl-1,2,3,4-tetrahydro-2,4-dioxopyrimidin-5-yl)-2-hydroxy-4-oxo-but-2-enoic acid (Figure 3, compound **22**). The compound was synthesized in our laboratory from readily available ethyl β-ketobutyrate [4].

Compound **22** and its difluoro analog **23** showed inhibition of both the 3′P and ST steps, although inhibition of the 3´P step was in the low µM level. The observed inhibition of both steps of integrase enzymology is somewhat unusual as other reported β-diketo acids were generally inhibitors of only the ST step (Table 1). Although the same active site residues appear to be involved in 3′-processing and ST, it is not clear whether the mechanism of binding and inhibition of HIV integrase by **22** is the same for 3′P in the cytoplasm and ST in the nucleus, *i.e.*, interaction with the DDE motif (Asp64, Asp116 and Glu152) and other proximal amino acid residues and possible sequestration of critical metal cofactors in the catalytic site. Participation of the carbonyl group at the 4-position of uracil in the binding of this inhibitor to the active site is suggested by our docking experiments [49]. Some support for the contribution of the uracil ring in the inhibition of both steps of HIV integrase comes from the inhibition data for **1 (L-708,906)**, which lacks the nucleobase scaffold. This compound **1** exhibited an IC_50_ of > 1000 µM for the 3′P step and 0.10 to 3.5 μM for the ST step. The EC_50_ was 5.5 µM in MT-4 cells [26,34].

**Figure 4:**
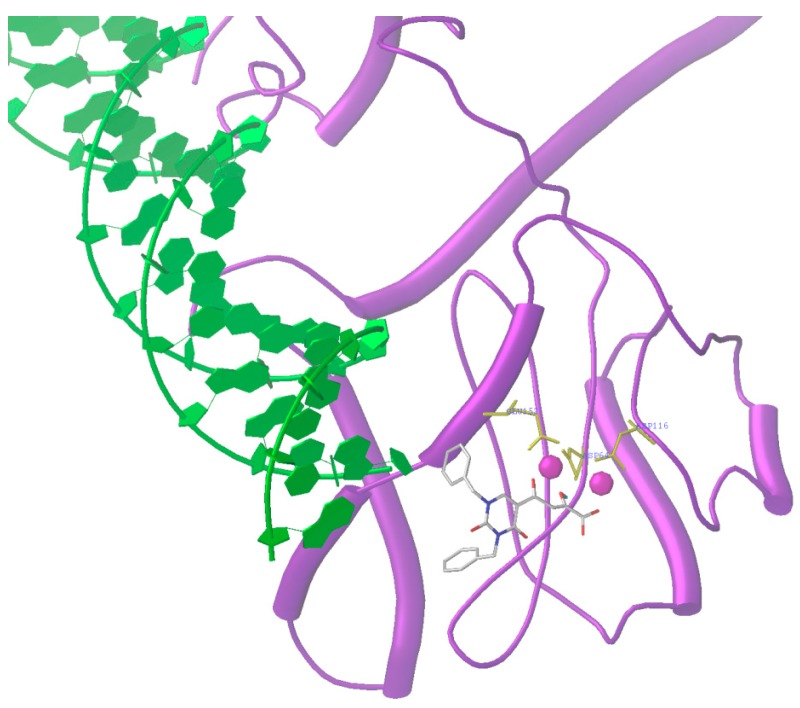
Docking of HIV integrase inhibitor, (**22**), with HIV integrase-viral DNA complex. The De Luca model of the integrase-LTR complex was used for docking. The DDE catalytic triad (Asp64, Asp116 and Glu152) and two Mg^2+^ ions (spheres) are shown. The compound binds to integrase by coordinating with the metal ions present in the catalytic core. The docked position is also stabilized by hydrogen bonding between Asp64 and the enol hydroxyl group [49].

Figure 4 shows our docking results of inhibitor **22** with HIV integrase-viral DNA complex. Inhibitor **22** functions by competing with the LTR of viral DNA for the active site of integrase.

The activity of compound **22** requires special mention (Table 3). The *in vitro* anti-HIV studies against HIV-1 isolates in PBMC showed that compound **22** (highest test concentration = 200 μM) was extremely active with EC_50_ values in the nM range. Cell viability data showed low cellular cytotoxicity (CC_50_ > 200 μM), which resulted in excellent antiviral efficacy data [therapeutic indices, TI = CC_50_/EC_50_ were > 4000 (HIV-1_TEKI_) and > 10,000 (HIV-1_NL4-3_)]. The EC_90_ data for **22**, which were in the low μM range, were also compelling. The control compound, AZT (highest test concentration = 1 μM), gave therapeutic indices of >7143 (HIV-1_TEKI_) and >5556 (HIV-1_NL4-3_). Comparison of the anti-HIV therapeutic indices of **22** and **11** (**S-1360)** in PBMC showed that the TIs for **22** were well over an order of magnitude greater than those of **11** (**S-1360)** (Table 1 and Table 3).

**Table 3:** Some representative examples of inhibitors of wild type HIV-1 integrase with anti-HIV activity from our laboratory [4,49,50,51].

Compound	Integrase Inhibition Data IC_50_ (μM) *	Cell Lines & HIV Isolates	EC_50_ *	CC_50_ *	TI *
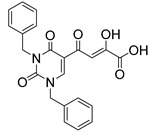 **22**	3.7 (3′P) 0.2 (ST) Mn^2+^ Assay	PBMC HIV-1_TEKI_ HIV-1_NL4-3_	50 nM <20 nM	>200 μM >200 μM	>4000 >10,000
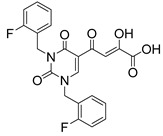 **23**	4.1 (3′P) <0.6 (ST) Mn^2+^Assay	GHOST X4/R5 HIV-1_TEKI_ HIV-1_NL4-3_	0.85 μM 0.24 μM	>200 μM >200 μM	>235 >833
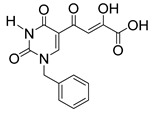 **24**	10 (3′P) 0.5 (ST) Mn^2+^ Assay	PBMC HIV-1_TEKI_ HIV-1_NL4-3_	2.63 μM 12.2 μM	>200 μM >200 μM	>76 >16.4
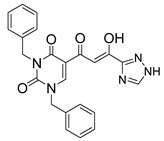 **25**	17 (3′P) 3 (ST) Mn^2+^ Assay	PBMC HIV-1_TEKI_ HIV-1_NL4-3_	2.50 μM 2.20 μM	>79.4 μM >79.4 μM	31.8 36.1
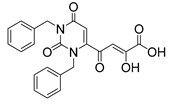 **26**	62 (3′P) 51 (ST) Mn^2+^ Assay	PBMC HIV-1_TEKI_ HIV-1_NL4-3_	151 μM 24.8 μM	>200 μM >200 μM	>1.3 >8.1
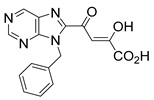 **27**	100 (3′P) 10 (ST) Mn^2+^ Assay	GHOST X4/R5 HIV-1_TEKI_ HIV-1_NL4-3_	5.26 μM 7.64 μM	97.7μM 97.7 μM	18.6 12.8

* IC_50_ is the concentration for 50% inhibition of the specified step of integrase activity. EC_50_ is the concentration for 50% inhibition of virus replication. CC_50_ is the concentration to reduce cell viability by 50%. TI (therapeutic index) is the ratio of CC_50_ to EC_50_.

### 2.6. Diketo Phosphonic Acids with Nucleobase Scaffolds

**Figure 5:**
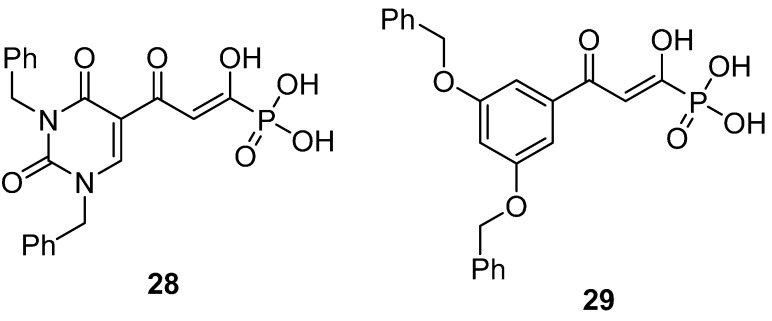
Structures of two phosphonic acid isosteres of compound **22** and **1** (**L-708,906)**.

Phosphonic acids have been viewed commonly as mimics of carboxylic acids, particularly with reference to biological activity. For example, amino phosphonic acids, isosteres of amino acids, reveal diverse biological properties [52]. With this concept in mind, we designed the β-diketo phosphonic acids, **28** and **29**, the phosphorus-based isosteres of compounds **22** and **1** (**L-708,906)** (Figure 5).

**Scheme 1:**
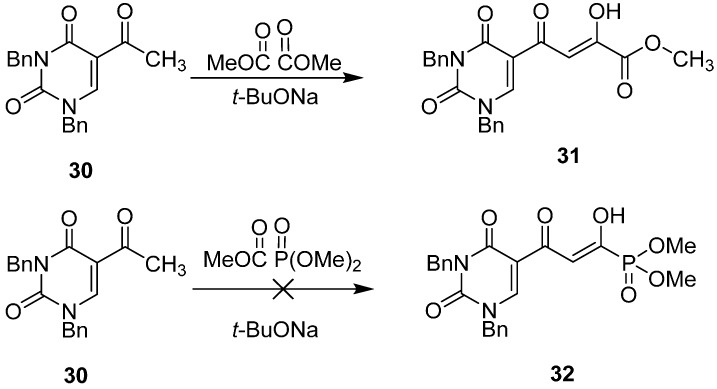
The difference in reactivity of **30** with dimethyl oxalate compared to trimethyl phosphonoformate.

The synthetic methodology to produce these compounds also needed to be developed. Although the β-diketo acid **22** (Table 3) was synthesized using as the key step, the condensation of acetyl uracil **30** with dimethyl oxalate under basic conditions [4], the related reaction with **30** and trimethyl phosphonoformate was unsuccessful (Scheme 1). The reason for this may be the instability of the C(O)-P(O) bond of trimethyl phosphonoformate under basic conditions [53].

**Scheme 2:**
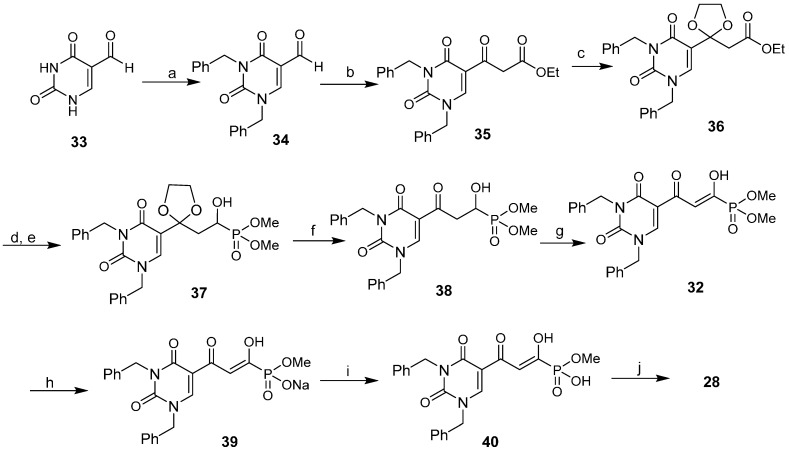
Synthesis of integrase inhibitor **28**. *Reagents and conditions*: (**a**) BnBr/DMF/K_2_CO_3_ (89%); (**b**) N_2_CHCO_2_Et/SnCl_2_/ CH_2_Cl_2_; (**c**) Ethylene glycol/CH(OEt)_3_/PTS (**b** and **c** 28%); (**d**) Dibal-H/toluene/−78 °C; (**e**) HP(O)(OMe)_2_/Et_3_N/MeOH (d and e 50%); (**f**) PTS/acetone/H_2_O (65%); (**g**) Dess-Martin periodinane/CH_2_Cl_2_; (**h**) NaI/acetone (**g** and **h** 67%); (**i**) Dowex50Wx8-100/MeOH; (**j**) NaI/acetone/reflux (**i** and **j** 51%).

In the alternative and successful route [50] (Scheme 2), 5-formyl uracil **33** was benzylated to give **34** (89%). Compound **34** was condensed with ethyl diazoacetate in the presence of tin (II) chloride [54] to afford **35**, which was transformed into its protected form **36** (28% yield from **34**). Reduction of **36** to the corresponding aldehyde with diisobutylaluminium hydride at −78 °C, followed by a Pudovik reaction with dimethyl phosphite and triethylamine, proceeded to form the phosphonate **37** (50% for two steps). The ketal group of **37** was deprotected to produce the key intermediate **38**, which was purified by HPLC (65% yield) and fully characterized by multinuclear NMR and HRMS data [49]. Dess-Martin oxidation of **38** produced diketone **32**, which exists largely in the enolic form. Compound **32** was deprotected by stirring with NaI in acetone for three days [55]. The resulting precipitate was collected and washed with acetone to afford the sodium methyl phosphonate **39** (67% yield). However, compound **39** could not be further deprotected with NaI, even under acetone reflux conditions, possibly because of the presence of the negative charge on the phosphonyl group and the poor solubility of **39** in acetone [55]. The problem was circumvented by conversion of **39** to its protonated form by ion-exchange chromatography with Dowex 50 × 8 in methanol to afford **40**. When compound **40** was heated under reflux with five equivalents of NaI in acetone for 24 h and the resulting crystalline precipitate was collected and washed with acetone, the target phosphonate **28** (as its monosodium salt) was produced in a highly purified form (51% yield). Compound **29** was synthesized by a similar procedure [49].

The anti-HIV activity of **28** and **29** are summarized in Table 4 [50]. Compound **28** was not an inhibitor of the 3′P step of HIV integrase (IC_50_ > 333 μM) and it was a weak inhibitor of the ST step (IC_50_ 20.2 μM). The ST integrase inhibition activity of **28** is in sharp contrast to that of compound **22** (IC_50_ 200 nM) [4]. As shown in Table 4, the *in vitro* anti-HIV studies of compound **28** revealed that it had low activity with EC_50_ values in the μM range. Cell viability data for **28** showed only mild cellular cytotoxicity at the highest test concentrations. The TI values were >50 (HIV-1_TEKI_) and >62 (HIV-1_NL4-3_)]. Compound **29** was significantly less active and more toxic [TI = 5.8 (HIV-1_TEKI_) and 4.0 (HIV-1_NL4-3_)].

**Table 4:** Antiviral data for compounds shown in Figure 5 on HIV-1 infected PBMC [50].

Compounds	HIV-1 Isolate (Cell Line)	EC_50_ *	CC_50_ *	TI *
**28**	HIV-1_TEKI_ (PBMC)	4.0 μM	>200 μM	>50
	HIV-1_NL4-3_(PBMC)	3.2 μM	>200 μM	>62
**29**	HIV-1_TEKI_ (PBMC)	8.1 μM	46.9 μM	5.8
	HIV-1_NL4-3_(PBMC)	11.8 μM	46.9 μM	4.0

* EC_50_ is the concentration for 50% inhibition of virus replication. CC_50_ is the concentration to reduce cell viability by 50%. TI (therapeutic index) is the ratio of CC_50_ to EC_50_.

### 2.7. Diketo Carboxylic Acids Constructed on Pyridinone Scaffold

**Figure 6:**
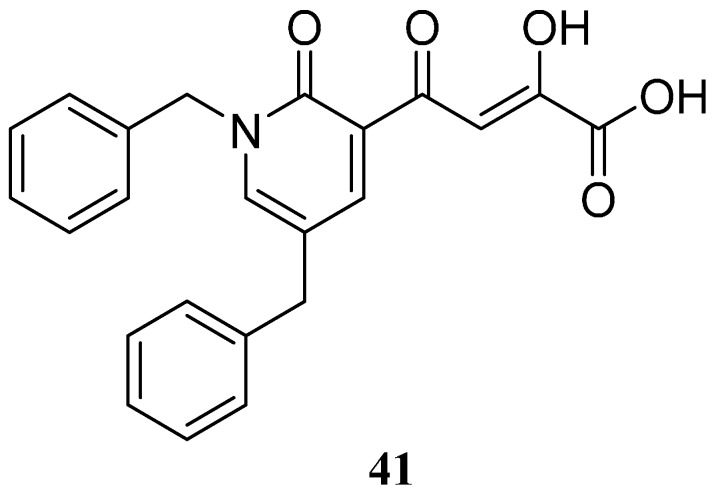
Structure of parent diketo acid constructed on a pyridinone scaffold.

To explore whether an analog of compound **22** with a pyridinone scaffold would possess more significant anti-HIV activity, we synthesized 4-(1,5-dibenzyl-1,2-dihydro-2-oxopyridin-3-yl)-2-hydroxy-4-oxobut-2-enoic acid (**41**, Figure 6) and discovered that it was an effective inhibitor of the integrase ST step, displaying an IC_50_ of 70 nM. Using compound **41** as a starting point, we undertook lead optimization studies [51,56] focusing on the structure of the modified nucleobase scaffold (*i.e.*, the pyridinone ring) and the nature of the substituents on the scaffold (diketo acid component as well as the hydrophobic benzyl groups), that were all deemed to be critical for integrase inhibitory activity.

Thus, in the next phase of the structure–activity studies, we examined the effects of various substituents on the phenyl rings to evaluate their effects on the inhibition of the integrase ST step. Considerable variations in the ST inhibitory activity for these compounds were seen and the IC_50_ data varied from <10 nM to >1500 nM. In general, the fluoro substitution IC_50_ data were the most interesting. Among this entire group of fluorinated compounds, the difluoro, trifluoro and tetrafluoro substituted compounds exhibited ST inhibitory IC_50s_ that were in the lower than 10 nM range [56], which represented a significant improvement over the lead compound **41** (IC_50_ 70 nM). Within this group of fluorinated compounds, the trifluoroaryl (*o-* and *o*,*p*) and tetrafluoroaryl (*o*,*p* and *o*,*p*) substituted analogs (involving both phenyl rings) were the most active in terms of the integrase IC_50_ and IC_90_ data (≤6 nM and <100 nM, respectively). While the detailed reason for the increase in inhibitory potency with appropriate fluorine substitution is not fully understood, hydrophobic and/or electrostatic interactions appear to be contributing factors [56,57,58,59].

In the next stage of lead optimization, we investigated the antiviral cell culture data for these compounds. The results are summarized in Table 5. They indicate that the anti-HIV-1 EC_50_ data were largely in the 1000–3000 nM range. However, two compounds emerged from these studies that exhibited anti-HIV EC_50_ values of 500 nM or less. They were 4-(1,5-bis(2,4-difluorobenzyl)-2-oxo-1,2-dihydropyridin-3-yl)-4-hydroxy-2-oxobut-3-enoic acid (**51**) and 4-(5-(2,4-difluorobenzyl)-1-(2-fluoro-benzyl)-2-oxo-1,2-dihydro-pyridin-3-yl)-2-hydroxy-4-oxobut-2-enoic acid (**96)**. However, their ST inhibition IC_50_ data were 5.5 ± 1.5 nM and 6 ± 3 nM, respectively [56]. The eventual selection of these compounds as the key compounds for further investigation is discussed in the prodrug section.

**Figure 7:**
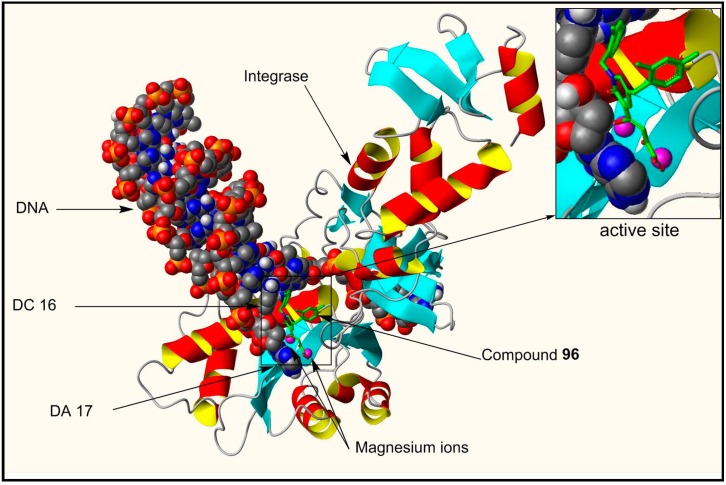
Docking pose of compound **96** (stick model in green) within the active site of modeled HIV-1integrase intasome. The inset illustrates the chelation of the two magnesium metals (indicated as purple spheres) with the inhibitor. The docking picture also shows ring-stacking interactions of compound **96** with DC 16 (deoxycytidine 16) as well as with DA 17 (deoxyadenosine 17) within the active site. Some of the key amino acid residues in integrase interacting with compound **96** are as follows: A/Asp64, A/Asp116, A/Tyr143, A/Gln144, A/Pro145, A/Gln148, A/Gly149, and A/Glu152. Molecular modeling of the crystal structure of prototype foamy virus (PFV) integrase intasome (PDB code 3OYA) [15,60] with compound **96** docked within the catalytic site was achieved by using the Surflex-Dock package within Sybyl-X [Sybyl-X1.3 (winnt_os5x) version] (Tripos, St. Louis, MO, USA, 2011).

The proposed mechanism of inhibition of integrase by compound **96** is depicted in Figure 7. Binding of **96** to the intasome involves processed viral DNA, two magnesium ions in the integrase active site and amino acids residues Asp64, Asp116, Tyr143, Gln144, Pro145, Gln148, Gly149, and Glu152.

**Table 5:** *In Vitro* anti-HIV data for analogs of compound **41** [56]. 
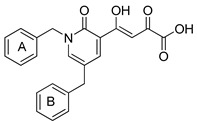

Compound	Aryl Ring A			Aryl Ring B			EC_50_ (nM) *^a,b^*
	*o*	*m*	*p*	*o*	*m*	*p*	
**41**	H	H	H	H	H	H	2100
**42**	F	H	H	H	H	H	900
**43**	H	H	F	H	H	H	1000
**44**	F	H	F	H	H	H	600
**45**	F	H	H	F	H	H	700
**46**	H	H	OMe	H	H	H	1800
**47**	H	H	F	H	H	F	800
**48**	H	H	Me	H	H	F	1600
**49**	H	Cl	F	H	H	H	800
**50**	H	H	OMe	H	H	OMe	>5000
**51**	F	H	F	F	H	F	300
**52**	H	F	H	H	H	H	2000
**53**	H	Cl	H	H	Cl	H	1100
**54**	H	Cl	F	H	Cl	H	1200
**55**	F	H	H	H	H	F	700
**56**	H	Cl	F	H	Cl	F	2400
**57**	H	H	Me	H	Cl	F	>5000
**58**	H	H	Me	H	Cl	H	2500
**59**	H	H	Me	F	H	H	700
**60**	H	H	F	H	Cl	F	>5000
**61**	F	Cl	F	H	H	H	600
**62**	H	H	F	H	Cl	H	1400
**63**	2,6-diF	H	H	H	H	F	1400
**64**	H	H	F	F	H	H	800
**65**	F	Cl	H	F	Cl	H	2400
**66**	H	H	Cl	H	Cl	H	2500
**67**	H	Cl	H	H	Cl	H	1400
**68**	F	Cl	H	H	Me	H	1400
**69**	F	H	F	F	H	H	600
**70**	H	Me	H	H	Cl	F	2200
**71**	F	Cl	H	H	H	H	700
**72**	F	5-Cl	H	H	H	H	900
**73**	F	Cl	H	H	Cl	H	1400
**74**	F	H	F	H	Cl	F	1400
**75**	H	Cl	H	H	H	F	1200
**76**	H	Cl	H	F	H	H	700
**77**	2,6-di-F	H	H	H	Cl	F	5100
**78**	2,6-di-F	H	H	H	H	H	1500
**79**	H	H	H	F	H	F	800
**80**	F	H	H	H	Cl	F	2200
**81**	H	F	H	F	H	H	1900
**82**	F	H	H	H	Cl	H	1900
**83**	F	F	H	H	H	H	900
**82**	H	Me	H	F	H	H	900
**85**	F	F	H	H	H	F	700
**86**	H	H	CN	F	H	H	1100
**87**	F	F	H	F	H	H	800
**88**	H	F	H	H	H	F	1000
**89**	F	H	F	Cl	H	H	800
**90**	H	Cl	H	F	Cl	H	1500
**91**	H	H	F	F	H	F	600
**92**	H	Me	H	F	H	F	700
**93**	H	H	H	H	COOH	H	>5000
**94**	H	H	H	Me	H	F	1300
**95**	H	H	F	Me	H	F	1000
**96**	F	H	H	F	H	F	500
**97**	F	H	H	Me	H	F	1300
**98**	H	Cl	F	F	H	H	790
**99**	F	H	F	H	H	F	420
**100**	F	Cl	H	F	H	H	500

*^a^* EC_50_ values are the average of three determinations. Standard deviations for the EC_50_ are within 31% of the average. *^b^* EC_50_ = concentration for 50% inhibition of the replication of HIV-1.

**Scheme 3:**
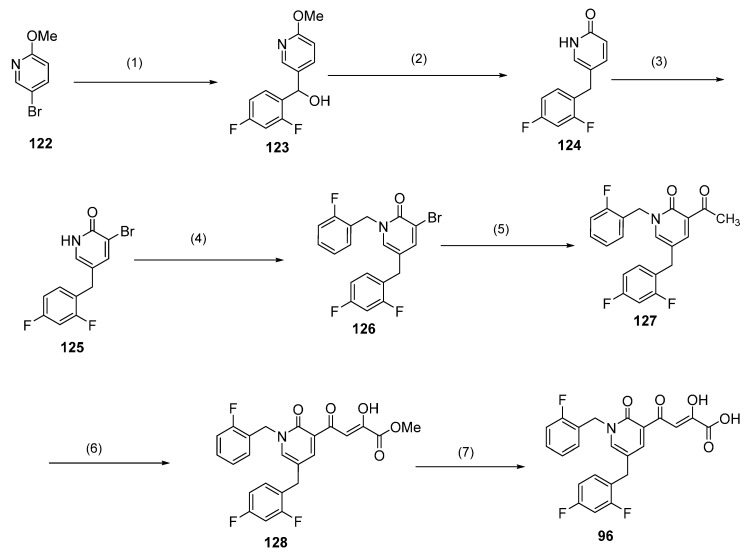
Methodology developed for the synthesis of integrase inhibitor **96**. *Reagents and conditions*: (**1**) **a**. *n*-BuLi/TBME, **b**. 2,4-difluorobenzaldehyde (93%); (**2**) TMSCl/NaI/TES/TFA/MeCN (94%); (**3**) NBS/CHCl_3_ (87%); (4) **a**. NaH/DMF, **b**. 2-FluoroBnBr/DMF (90%); (**5**) **a**. 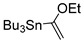, (Ph_3_P)_2_PdCl_2_/DMF, **b**. 1N HCl (91%); (**6**) **a**. *t*-BuONa/THF, MeO_2_CCO_2_Me, **b**. 1N HCl (77%); (**7**) 1N HCl/dioxane (78%).

The aforementioned anti-HIV active integrase inhibitors would not have been discovered without the development of a general methodology for their syntheses, which is exemplified with the example of compound **96** in Scheme 3. Only seven steps (aromatic nucleophilic addition, demethylation/deoxygenation, radical bromination, benzylation, palladium-catalyzed cross-coupling, Claisen condensation and acid-catalyzed hydrolysis) [61,62,63,64,65,66,67] were required for the total synthesis of **96** from commercially available 5-bromo-2-methoxypyridine (**122**). The overall yield of **96** from **122** was 37% [56].

## 3. Prodrug Design, Synthesis and Antiviral Evaluation

### 3.1. Anti-HIV Diketo Acid Prodrug Development

**Scheme 4:**
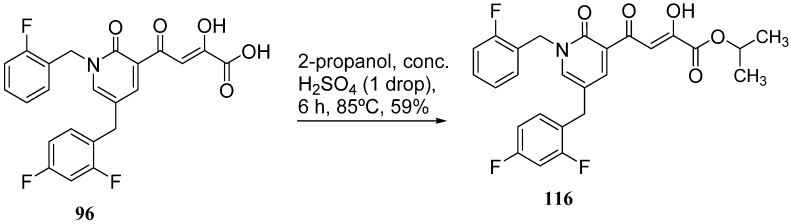
General synthesis of pro-drugs exemplified with **116**, the pro-drug of compound **96**.

Cell culture antiviral data of compound **96** revealed a substantial disconnect of almost two orders of magnitude between its *in vitro* anti-HIV-1 activity data (EC_50_ 500 nM, MAGI cells) and its ST inhibition data (IC_50_ 6 nM). In general, for HIV-1 integrase inhibitors, there is normally a realistically good correlation between ST IC_50_ and cell culture EC_50_ [3,5]. It was concluded by us that the lack of correlation between the IC_50_ and EC_50_ data for compound **96**, as well as other compounds, including **51** (Table 5), might be associated with low cellular permeability of the inhibitors, because they would be expected to be in the ionized state under physiological conditions. Therefore, we investigated prodrug esters of these compounds in order to verify and to find a solution to this problem [56]. The isopropyl ester pro-drug, **116**, was easily synthesized from compound **96** through acid-catalyzed esterification with 2-propanol (Scheme 4).

Comparison of the cLog P values for compound **96** and its isopropyl ester **116** of 2.38 and 4.24, respectively, suggested that compound **116** is significantly less polar than compound **96** anion and thus would be expected to be cellularly more permeable. Our expectation was that this would be reflected in the cell-based anti-HIV data and this was convincingly confirmed. The pro-drug **116** improved the anti-HIV activity of **96** and had an EC_50_ of 9 ± 4 nM (MAGI cells), lowering it to 1.8% of the EC_50_ of the diketo acid **96** [56]. This was the best *in vitro* activity achieved of all of the prodrugs studied in this work (EC_50s_ ranged from 9 nM to 46 nM for isopropyl ester prodrugs). The isopropyl ester prodrug of **51** (Table 6, compound **109**), although less active than compound **116**, nevertheless exhibited an EC_50_ of 46 ± 18 nM (MAGI cells). The overall performance of the assay was validated by the MOI-sensitive positive control compound, raltegravir, which exhibited the expected level of antiviral activity (EC_50_ 6 nM) (see Table 7) [7,56]. Cell viability data for **116** showed only low toxicity at higher test concentrations (CC_50_ = 135 ± 7 µM, CC_90_ > 200 µM), although a CC_90_ was not reached at the highest test concentration of 200 µM. Also of significance was the fact that the EC_50_ and EC_90_ data for the prodrug **116** of 9 nM and 94 nM, respectively, had exceptionally strong correlation with the ST inhibition IC_50_ and IC_90_ data of 6 nM and 97 nM, respectively, for the corresponding diketo acid **96**. The therapeutic (selectivity) index TI (CC_50_/EC_50_) of compound **116** was 14,600.

It is also of significance to indicate that the isopropyl ester, **116**, was not a strong inhibitor of HIV integrase in enzymatic studies (IC_50_ > 475 nM), suggesting that the anti-HIV activity of **116** was most likely the result of its cellular uptake and subsequent hydrolysis in the cell to produce the cellularly-active anti-HIV compound **96**. Consistent with this conclusion was our observation that compound **116** underwent rapid hydrolysis in human liver microsomes to produce compound **96** (100% conversion in <15 min) [56].

**Table 6:** Pro-drug SAR studies: Examples of ester pro-drugs [56].

Prodrug Derivatives of Integrase Inhibitors	Prodrug Anti-HIV EC_50_ (MAGI Cell) *	Diketo Acid Anti-HIV EC_50_ (MAGI Cell) *
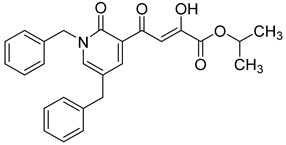 **101**	300 nM	2100 nM (Compound **41**)
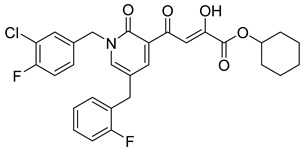 **102**	>5000 nM	710 nM (Compound **98**)
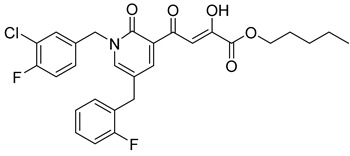 **103**	>5000 nM	710 nM (Compound **98**)
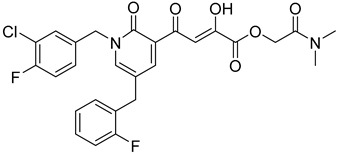 **104**	1900 nM	710 nM (Compound **98**)
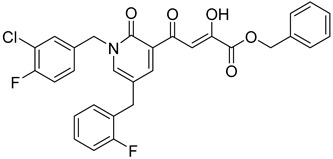 **105**	5300 nM	710 nM (Compound **98**)
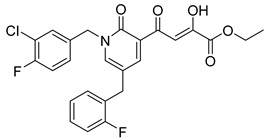 **106**	1600 nM	710 nM (Compound **98**)
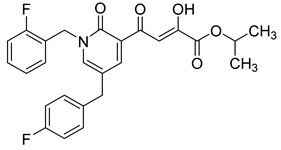 **107**	40 nM	700 nM (Compound **55**)
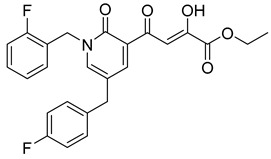 **108**	3900 nM	700 nM (Compound **55**)
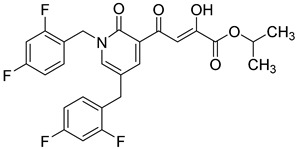 **109**	50 nM	300nM (Compound **51**)
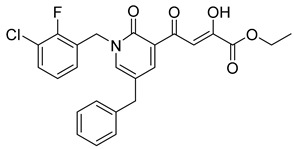 **110**	>5000 nM	700 nM (Compound **71**)
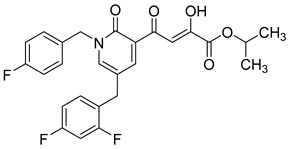 **111**	15 nM	600 nM (Compound**91**)
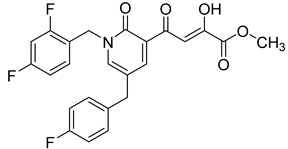 **112**	400 nM	420 nM (Compound **99**)
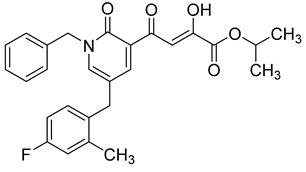 **113**	100 nM	1300 nM (Compound **94**)
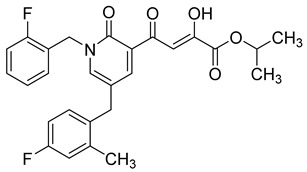 **114**	110 nM	1300 nM (Compound **97**)
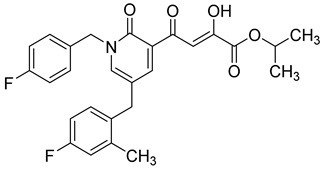 **115**	226 nM	1000 nM (Compound **95**)
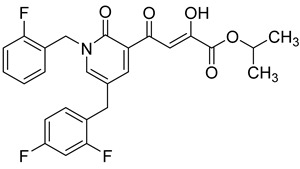 **116**	9 nM	500 nM (Compound **96**)
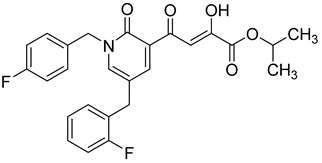 **117**	176 nM	800 nM (Compound **64**)
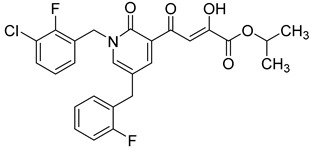 **118**	1200 nM	500 nM (Compound **100**)
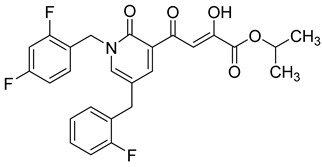 **119**	400 nM	600 nM (Compound **69**)
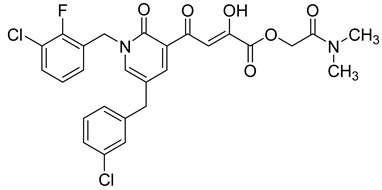 **120**	4400 nM	1400 nM (Compound **73**)
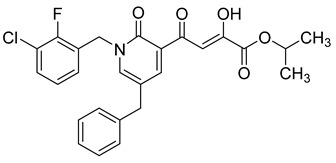 **121**	2700 nM	700 nM (Compound **71**)

* EC_50_ = concentration for 50% inhibition of the replication of HIV-1.

**Table 7:** Prodrug molecules of our diketo acids with significant antiviral activity.

Prodrug	Anti-HIV EC_50_ (MAGI Cell Data) *
**107**	40 nM
**109**	50 nM
**111**	15 nM
**116**	9 nM
**Raltegravir (positive control compound)**	6 nM

* EC_50_ = concentration for 50% inhibition of the replication of HIV-1.

### 3.2. Stability, Metabolism and CYP and UGT Drug Interaction Studies on Compound **96**

**Figure 8:**
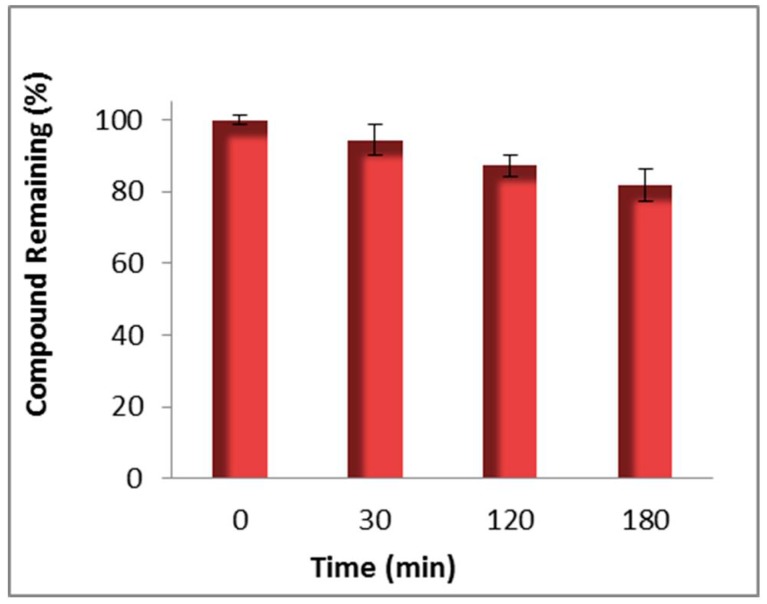
Stability of **96** in pooled human liver microsomes monitored by HPLC with UV detection. The error bars are standard deviation (±SD) from the mean of three determinations.

Stability studies on compound **96** in pooled human liver microsomes were carried out by pre-incubation, initiation with NADPH, further incubation at 37 °C, quenching of samples with cold acetonitrile at selected time intervals, centrifugation and quantitative analysis of the supernatant by HPLC [56,68,69,70]. The conclusion from these studies was that integrase inhibitor **96** was relatively stable in human liver microsomes. The *in vitro* half-life was >> 3 h, as 80% of compound **96** was still present after the 3 h incubation in human liver microsomes (Figure 8). A slowly produced key metabolite was identified by HPLC and HRMS data to be the product of retro-Claisen cleavage of the diketo group of **96** to produce the acetyl pyridinone **127** (see Scheme 3).

The *in vitro* drug interaction profile involving kinetic studies with key cytochrome P450 (CYP) isozymes [69,70,71] and appropriate substrates in pooled human liver microsomes with varying concentrations of **96** showed that compound **96** was not an inhibitor of CYP3A4 and CYP2D6 isozymes and was a very weak inhibitor of the CYP2C8 isozyme (Table 8). These key isozymes account for a total of over 80% of drugs that are metabolized by different CYP isoforms. In addition, compound **96** did not exhibit any activation of these CYP isozymes. Thus, our studies suggest that this integrase-based, anti-HIV compound is anticipated to have a favorable drug interaction profile with respect to key CYP isozymes.

**Table 8:** IC_50_ data for inhibition of key cytochrome P450 isozymes for compound **96** [56].

CYP450 Isozyme	Substrate (Stock Solution)	Conc. (µM )	Protein (mg/mL)	Incubation (min)	IC_50_ Data (µM) *
CYP3A4	Testosterone (50 mM )	100	0.3	30	>200 µM
CYP3A4	Triazolam (50 mM)	200	0.4	30	>200 µM
CYP2D6	Dextromethorphan (50 mM)	200	2.0	60	>200 µM
CYP2C8	Amodiaquine (5 mM)	200	0.4	30	>65 µM

* IC_50_ is the concentration for 50% inhibition of the specified CYP isozyme from kinetic data.

Because isozymes of uridine 5′-diphospho-glucuronosyltransferase (UGT) also play an important role in determining drug-drug interactions, we investigated the substrate activity of **96** towards key human UGTs [68,72,73]. Compound **96** was not a substrate for the following key UGT isozymes: 1A1, 1A4, 1A6, 1A9 and 2B7.

## 4. Conclusions

In summary, the retroviral enzyme, HIV integrase, catalyzes the incorporation of HIV DNA into human chromosomal DNA. Inhibitors of this point of “no-return” in HIV infection are of considerable significance in anti-HIV drug discovery. This review article has focused on the issue of the lack of correlation between integrase ST enzymology data (IC_50_) and the corresponding cellular anti-HIV EC_50_ data for beta-diketo acid inhibitors of integrase. Comprehensive analyses of these data revealed that the disconnect appeared to be a problem associated with drug cellular permeability. This conclusion was convincingly supported through the synthesis and anti-HIV activity studies of prodrugs of strand transfer inhibitors of integrase. In addition, it was also confirmed that these prodrugs were rapidly and quantitatively hydrolyzed in cells to the active compounds.
